# Supplementary material for: The evolution of reproductive strategies in turtles
Source: PeerJ. 2022 Mar 11;10:e13014. doi: 10.7717/peerj.13014 (PMC8919852; doi:10.7717/peerj.13014)
Supplement: Supplemental Information 2 — Methods description and PRISMA workflow diagram. [file peerj-10-13014-s002.docx]

**Appendix S2 – Data acquisition + PRISMA diagram**

We used *Google scholar* database to perform an electronic search using different combinations of the key words “Egg size”, “turtle reproduction”, “breeding”, “nest”, “clutch size”, “egg width”. Studies from all dates were considered as evolutionary characteristics of species do not usually change within the relevant time for a literature search. Only full‐text reports in English, Spanish and Portuguese were considered. Study eligibility was assessed by one investigator. A secondary search was conducted on the reference list of these publications as well as on the list of publications that have cited the previous accessed one. The search continued until the limit of four articles containing information on the same ecological data for each species.

We used the PRISMA checklist (Moher et al. 2011) as a guide for quality reporting of our review.

Data used was based on a combination of the information available (e.g., smallest and biggest clutch sizes reported, even if from different sources) or based on the most common attribution for each species (e.g., species that are found both in land and water but most commonly in water were addressed with this kind of habitat for the related analysis). Data from captivity was considered as the characteristics of interest are mostly supposed to be inheritable and we considered possible bias to be irrelevant. Selected data is available at appendix 1 of the supplemental material. Any other specific bias considered is described on the “comments” column.

**Identification of studies via databases and registers**

Records removed *before screening*:

Duplicate records removed (n = 0)

Records marked as ineligible by automation tools (n = 0)

Records removed for other reasons (n = 0)

Records identified from:

Databases (n = 1)

Registers (n ~ 98)

**Identification**

Records screened

(n ~ 98)

Records excluded

(n ~ 17)

Reports sought for retrieval

(n = 81)

Reports not retrieved

(n = 0)

**Screening**

Reports assessed for eligibility

(n = 81)

Reports excluded for not containing relevant information:

(n = 24)

Studies included in review

(n = 57)

**Included**

**References**

Alvarenga, C.C.E.D., 2006. Aspectos da biologia reprodutiva de Rhinemys rufipes (Spix, 1824)(Chelidae, Testudines) na Reserva Florestal Adolpho Ducke, Amazonas, Brasil.

Bager et al. 2016. Morphology and sexual dimorphism of Acanthochelys spixii (Testudines, Chelidae) in Brazil.

Böhm, S., 2011. Ecology of the chelid turtles Platemys platycephala, Mesoclemmys gibba and Mesoclemmys nasuta in French Guyana (Doctoral dissertation, uniwien).

Bonin, F., Devaux, B. and Dupré, A., 2006. Turtles of the World. JHU Press.

Booth, D.T., Burgess, E., McCosker, J. and Lanyon, J.M., 2004, December. The influence of incubation temperature on post-hatching fitness characteristics of turtles. In International Congress Series (Vol. 1275, pp. 226-233).

Booth, D.T., 1998. Egg size, clutch size, and reproductive effort of the Australian broad-shelled river turtle, Chelodina expansa. Journal of Herpetology, 32(4), pp.592-596.

Booth, D.T., 2006. Influence of incubation temperature on hatchling phenotype in reptiles. Physiological and Biochemical Zoology, 79(2), pp.274-281.

Brooks, R.J., Shilton, C.M., Brown, G.P. and Quinn, N.W., 1992. Body size, age distribution, and reproduction in a northern population of wood turtles (Clemmys insculpta). Canadian Journal of zoology, 70(3), pp.462-469.

Bujes, C.S., 2010. Os Testudines continentais do Rio Grande do Sul, Brasil: taxonomia, história natural e conservação. Iheringia. Série Zoologia, 100(4), pp.413-424.

JoHN, C.A.N.N. and Legler, J.M., 1994. The Mary River tortoise: a new genus and species of short-necked chelid from Queensland, Australia (Testudines: Pleurodira). Chelonian Conservation and Biology, 1(2).

Cann, J., 1997. The northern yellow-faced turtle. Monitor (Victorian Herpetological Society, Melbourne), 9(1), pp.24-29.

Chinen, S., Lisboa, C.S. and Molina, F.B., 2004. Biologia reprodutiva de Hydromedusa tectifera em cativeiro (Testudines, Chelidae). Arq. Inst. Biol, 71, pp.401-403.

Clay, B.T., 1981. Observations on the breeding biology and behaviour of the long-necked tortoise, Chelodina oblonga. Journal of the Royal Society of Western Australia, 4(1), pp.27-32.

Congdon, J.D. and Gibbons, J.W., 1985. Egg components and reproductive characteristics of turtles: relationships to body size. Herpetologica, pp.194-205.

Congdon, J.D. and Loben Sels, R.C.V., 1991. Growth and body size in Blanding's turtles (Emydoidea blandingi): relationships to reproduction. Canadian Journal of Zoology, 69(1), pp.239-245.

Corazza, S.S. and Molina, F.B., 2004. Biologia reprodutiva e conservação ex-situ de Bufocephala vanderhaegei (Testudines, Chelidae). Arquivos do Instituto Biológico, 71, pp.407-409.

De Alvarenga, C.C.E., da Costa, B., Guterres, E., Reynolds, S. and Christian, K., 2014. Assessment of Chelodina mccordi current status and community awareness along the Lake Iralalaro, Timor-Leste.

Eiby, Y.A. and Booth, D.T., 2011. Determining optimal incubation temperature for a head-start program: the effect of incubation temperature on hatchling Burnett River snapping turtles (Elseya albagula). Australian Journal of Zoology, 59(1), pp.18-25.

Elgar, M. and Heaphy, L.J., 1989. Covariation between clutch size, egg weight and egg shape: comparative evidence for chelonians. Journal of Zoology, 219(1), pp.137-152.

Ewert, M.A., Jackson, D.R. and Nelson, C.E., 1994. Patterns of temperature‐dependent sex determination in turtles. Journal of Experimental Zoology, 270(1), pp.3-15.

Fagundes, C.K. and Bager, A., 2007. Ecologia reprodutiva de Hydromedusa tecifera (Testudines: Chelidae) no sul do Brasil. Biota Neotropica, 7(2), pp.0-0.

Famelli, S., Bertoluci, J., Molina, F.B. and Matarazzo-Neuberger, W.M., 2011. Structure of a population of Hydromedusa maximiliani (Testudines, Chelidae) from Parque Estadual da Serra do Mar, an Atlantic rainforest preserve in southeastern Brazil. Chelonian Conservation and Biology, 10(1), pp.132-137.

Famelli, S., Adriano, L.R., Pinheiro, S.C., Souza, F.L. and Bertoluci, J., 2014. Reproductive biology of the freshwater turtle Hydromedusa maximiliani (Chelidae) from southeastern Brazil. Chelonian Conservation and Biology, 13(1), pp.81-88.

Ferrara, C.R., Fagundes, C.K., Morcatty, T.Q. and Vogt, R.C., 2017. Quelônios Amazônicos: Guia de identificação e distribuição. Manaus, Brazil: Wildlife Conservation Society Brasil.

Fielder, D., Chessman, B. and GEORGES, A., 2015. Myuchelys bellii (Gray 1844)–Western Saw-shelled Turtle, Bell’s Turtle. Chelonian Research Monographs, pp.1-7.

Forero–Medina, G., Castaño–Mora, O.V., Cárdenas–Arévalo, G. and Medina–Rangel, G.F., 2013. Mesoclemmys dahli (Zangerl and Medem 1958). Dahl’s toad-headed turtle. Carranchina, Tortuga Montañera. Chelon Res Monogr, 5, pp.069-1.

Forsman, A. and Shine, R., 1995. Sexual size dimorphism in relation to frequency of reproduction in turtles (Testudines: Emydidae). Copeia, 1995(3), pp.727-729.

Freeman, A. and Branch, T.S., 2010. Saving a living fossil: identification and mitigation of threats to the conservation status of the freshwater turtle, Elseya lavarackorum. Unpublished report prepared for the Department of Environment, Water, Heritage and the Arts, Canberra.

Gaikhorst, G.S., Clarke, B.R., McPharlin, M., Larkin, B., McLaughlin, J. and Mayes, J., 2011. The captive husbandry and reproduction of the pink‐eared turtle (Emydura victoriae) at Perth Zoo. Zoo biology, 30(1), pp.79-94.

Georges, A., Guarino, F. and Bito, B., 2006. Freshwater turtles of the TransFly region of Papua New Guinea–notes on diversity, distribution, reproduction, harvest and trade. Wildlife Research, 33(5), pp.373-384.

Gheler-Costa, C., Lyra-Jorge, M.C. and Verdade, L.M. eds., 2016. Biodiversity in agricultural landscapes of southeastern Brazil. Walter de Gruyter GmbH & Co KG.

Hamann, M., Schauble, C.S., Emerick, S.P., Limpus, D.J. and Limpus, C.J., 2008. Freshwater turtle populations in the Burnett River. Memoirs of the Queensland Museum, 52, pp.221-232.

Highfield, A.C., 1996. Practical encyclopedia of keeping and breeding tortoises and freshwater turtles. Carapace Press.

Kennett, R., 1994. Ecology of two species of freshwater turtle, Chelodina rugosa and Elseya dentata, from the wet-dry tropics of northern Australia (Doctoral dissertation, University of Queensland).

Kennett, R., Fordham, D., Alacs, E., Corey, B. and GEORGES, A., 2014. Chelodina oblonga Gray 1841: Northern Snake-Necked Turtle. Chelonian Research Monographs, 5, pp.1-13.

Kuchling, G., 1989. Assessment of ovarian follicles and oviductal eggs by ultra-sound scanning in live freshwater turtles, Chelodina oblonga. Herpetologica, pp.89-94.

Legler, J. and Vogt, R.C., 2013. The turtles of Mexico: land and freshwater forms. Univ of California Press.

Lovich, J.E. and Ernst, C.H., 1989. Variation in the plastral formulae of selected turtles with comments on taxonomic utility. Copeia, pp.304-318.

McCord, W.P. and Thomson, S.A., 2002. A new species of Chelodina (Testudines: Pleurodira: Chelidae) from northern Australia. Journal of Herpetology, 36(2), pp.255-267.

MÉTRAILLER, S., 2006. Mesoclemmys gibba (Schweigger, 1812). In Turtles: Proceedings: International Turtle & Tortoise Symposium, Vienna 2002 (p. 338). Edition Chimaira.

Mocelin, M.A., Fernandes, R., Porto, M. and Fernandes, D.S., 2008. Reproductive biology and notes on natural history of the side-necked turtle Acanthochelys radiolata (Mikan, 1820) in captivity (Testudines: Chelidae). South American Journal of Herpetology, 3(3), pp.223-228.

Moher, D., Altman, D.G., Liberati, A. and Tetzlaff, J., 2011. PRISMA statement. Epidemiology, 22(1), p.128.

Moravec, J., 2017. First data on reproduction and hatchling morphology of Mesoclemmys heliostemma (MCCORD, JOSEPH-OUNI & LAMAR, 2001).

Páez, V.P., Morales-Betancourt, M.A., Lasso, C.A., Castaño Mora, O.V. and Bock, B.C., 2012. V. Biología y conservación de las tortugas continentales de Colombia. Serie Recursos Hidrobiológicos y Pesqueros Continentales de Colombia. Instituto de Investigación de Recursos Biológicos Alexander von Humboldt.

Prieto, Y., Bernardi, C.G., Rozycki, V.R. and Manzano, A.S., 2019. Chemical Composition of the Eggs of the Freshwater Turtle Phrynops hilarii (Chelidae: Testudines).

Pritchard, P.C. and Trebbau, P., 1984. The turtles of Venezuela. [Oxford, Ohio]: Soc. for the Study of Amphibians and Reptiles.

Mittermeier, A.R.R., 1983. Description of Phrynops williamsi, a new species of chelid turtle of the South American P. geoffroanus complex. Advances in Herpetology and Evolutionary Biology–Essays in Honor of EE Williams. Museum of Comparative Zoology, Cambridge, pp.58-73.

Rhodin, A.G.J., Ibarrondo, B.R. and Kuchling, G., 2008. Chelodina mccordi Rhodin 1994—Roti Island snake-necked turtle, McCord’s snake-necked turtle, kura-kura rote. Conservation Biology of Freshwater Turtle and Tortoises: A Compilation Project of the IUCN/SSC Tortoise and Freshwater Turtle Specialist Group. Chelonian Research Monographs, 5(1), pp.008-001.

Rueda-Almonacid, J.V., Carr, J.L., Mittermeier, R.A., Rodríguez-Mahecha, J.V., Mast, R.B., Vogt, R.C., Rhodin, A.G., de la Ossa-Velásquez, J., Rueda, J.N. and Mittermeier, C.G., 2007. Las tortugas y los cocodrilianos de los países andinos del trópico. Serie de guías tropicales de campo, 6, pp.412-423.

Santana, D.O., Marques, T.S., Vieira, G.H.C., Moura, G.J.B., Faria, R.G. and Mesquita, D.O., 2016. Mesoclemmys tuberculata (Luederwaldt 1926)—Tuberculate Toad-headed Turtle. Conservation Biology of Freshwater Turtles and Tortoises: A Compilation Project of the IUCN/SSC Tortoise and Freshwater Turtle Specialist Group. Chelonian Research Monographs, 5, pp.097-1.

Souza, F.L. and Abe, A.S., 2000. Feeding ecology, density and biomass of the freshwater turtle, Phrynops geoffroanus, inhabiting a polluted urban river in south‐eastern Brazil. Journal of Zoology, 252(4), pp.437-446.

Souza, F.L., 2004. Uma revisão sobre padrões de atividade, reprodução e alimentação de cágados brasileiros (Testudines, Chelidae). Phyllomedusa, 3(1), pp.15-27.

Thompson, M.B., 1983. The physiology and ecology of the eggs of the pleurodiran tortoise Emydura macquarii (Gray), 1831 (Doctoral dissertation).

Thomson, S., Amepou, Y.O.L.A.R.N.I.E., Anamiato, J. and Georges, A., 2015. A new species and subgenus of Elseya (Testudines: Pleurodira: Chelidae) from New Guinea. Zootaxa, 4006(1), pp.59-82.

Vanzolini, P.E., 2003. On clutch size and hatching success of the South American turtles Podocnemis expansa (Schweigger, 1812) and P. unifilis Troschel, 1848 (Testudines, Podocnemididae). Anais da Academia Brasileira de Ciências, 75(4), pp.415-430.

Vogt, R.C. and Bull, J.J., 1982. Temperature controlled sex-determination in turtles: ecological and behavioral aspects. Herpetologica, pp.156-164.

Zuffi, M.A.L., Odetti, F. and Meozzi, P., 1999. Body size and clutch size in the European pond turtle (Emys orbicularis) from central Italy. Journal of Zoology, 247(2), pp.139-143.
