# Supplementary material for: The evolution of reproductive strategies in turtles
Source: PeerJ. 2022 Mar 11;10:e13014. doi: 10.7717/peerj.13014 (PMC8919852; doi:10.7717/peerj.13014)
Supplement: Supplemental Information 4 — Same analyses were performed using a newer phylogeny (Thomson, Spinks & Shaffer, 2021). This phylogeny has smaller overlap with our dataset than the selected phylogeny for the manuscript (Pereira et al., 2017). [file peerj-10-13014-s004.docx]

| Model | σ ²1,1 | σ ² 1,2 | σ ² 1,3 | σ ² 2,1 | σ ² 2,2 | σ ² 2,3 | R1 | R2 | R3 | Log(L) | AIC |
| --- | --- | --- | --- | --- | --- | --- | --- | --- | --- | --- | --- |
| **common rates, common correlation** | **0.002** | **-** | **-** | **0.0001** | **-** | **-** | **0.474** | **-** | **-** | **193.88** | **-377.77** |
| different rates, common correlation | 0.0023 | 0.0017 | 0.001 | 0.0002 | 0.0001 | 0.0001 | 0.462 | - | - | 196.47 | -374.94 |
| common rates, different correlation | 0.0018 | - | - | 0.0001 | - | - | 0.375 | 0.557 | 0.361 | 194.78 | -375.57 |
| No common structure | 0.0023 | 0.0018 | 0.0007 | 0.0002 | 0.0002 | 0.0001 | 0.441 | 0.567 | 0.004 | 198.96 | -375.93 |

**Appendix S4 – Analyses with phylogeny from Thompson et al., 2021**

**Hierarchical models of evolutionary correlation among reproductive traits in turtles using the phylogeny by Thompson et al., (2021).** Model description, rates of correlation between egg size and three different clutch size groups (σ ² 1,*x*), rates of correlation between egg shape and three different clutch size groups (σ ² 2,*x*), correlation between egg size and egg shape, affected by different regimes of clutch size (R), log-likelihood (Log-L), and Akaike information criterion (AIC) for four multivariate Brownian evolution model fits to egg and clutch data. The best-supported model is highlighted in bold.


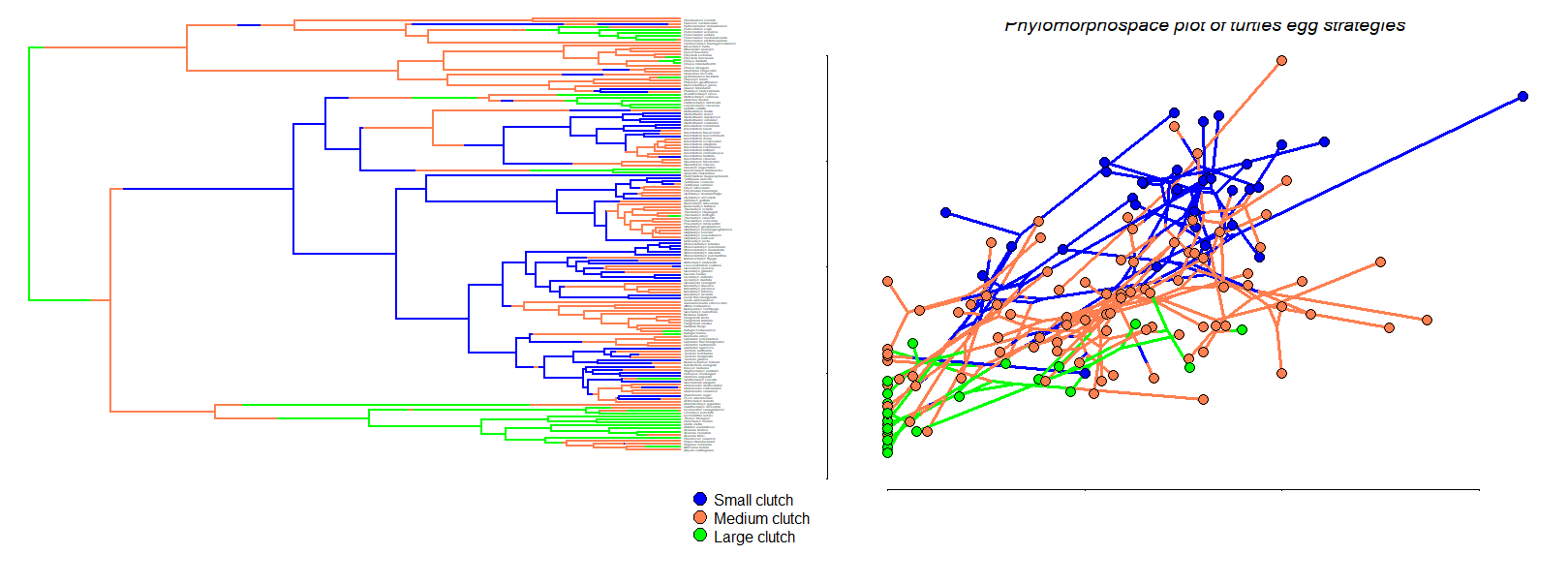


**Distribution of egg and clutch traits in the turtle phylogeny (Thompson et al., 2021).** Different clutch sizes were assigned to three different regimes (small, medium, and large) and mapped to the tree (A); Turtle phylogeny was plotted in a morphospace based on egg size and shape (B).
