# Supplementary figures and images for: The evolution of reproductive strategies in turtles
Source: PeerJ. 2022 Mar 11;10:e13014. doi: 10.7717/peerj.13014 (PMC8919852; doi:10.7717/peerj.13014)

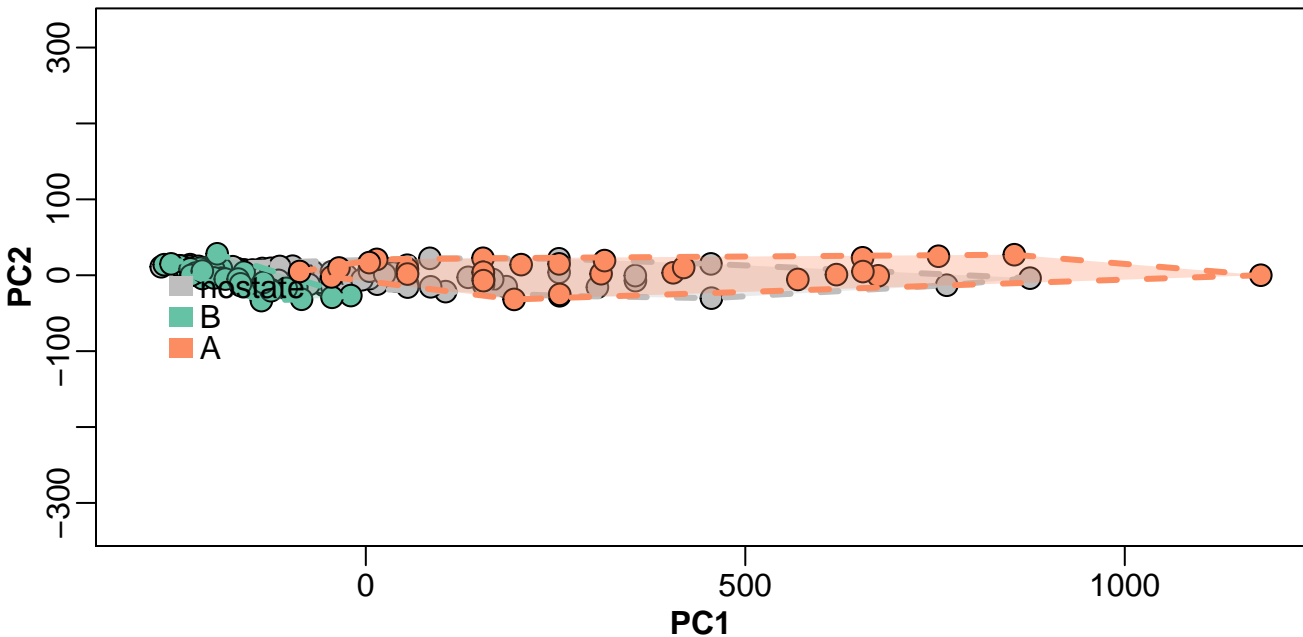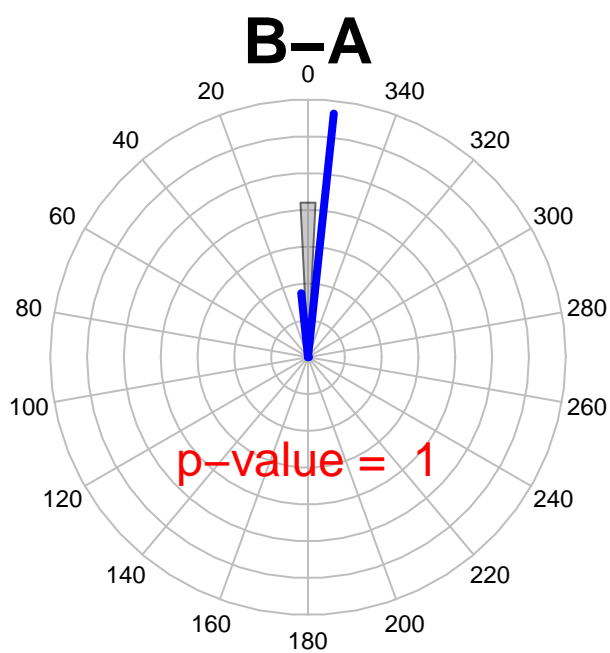

Supplement: Supplemental Information 5 — Colored convex hulls represent species belonging to different states: species that produce small and large clutches, containing over 30 eggs (state A) and up to four eggs (state B). The circular plot on the bottom represents the mean θreal angle between tested states (blue lines) and the range of θrandom angles (gray shaded area) of the same states. [file peerj-10-13014-s005.pdf]
